# Rethink context engineering using an attention-based architecture

**DOI:** 10.1038/s41598-026-43111-9

**Published:** 2026-03-07

**Authors:** Yiqiao Yin

**Affiliations:** 1 University of Chicago Booth School of Business, Chicago, USA; 2 Department of Statistics, Columbia University, New York, USA

**Keywords:** Engineering, Mathematics and computing

## Abstract

Accurate prediction of user actions is essential for optimizing digital platform workflows, enabling proactive recommendations, resource prefetching, and intelligent user assistance. Traditional Markov chain-based methods, though widely used for modeling sequential behavior, are fundamentally limited in capturing the complexity, long-range dependencies, and multi-objective nature of real-world user interactions. This paper introduces a multi-task attention-based transformer architecture for sequential API recommendation that addresses these gaps in robustness and generalizability. The core insight is that user behavior on enterprise platforms is driven by latent intent: users with different goals—such as executing a machine learning pipeline, conducting data analysis, managing user accounts, or generating quick visualizations—exhibit systematically different sequential patterns across functional API categories. Our framework exploits this structure through a shared transformer encoder backbone that produces a unified representation of the user’s action history, which is then decoded by three task-specific prediction heads operating simultaneously. The primary head predicts the next API action from a probability distribution over all available endpoints; an auxiliary goal classification head infers the user’s underlying session objective from the observed action sequence alone; and a session boundary detection head estimates the probability that the user is about to conclude their session. During inference, only the sequence of prior API calls is required as input—the model jointly infers what the user will do next, what they are trying to accomplish, and whether they are about to leave, all from the observed behavioral trace. Leveraging a large-scale simulated behavioral dataset encompassing 2, 000 user sessions and 20, 000 API calls across 100 APIs organized into 10 functional categories, with 4 distinct session goal types governing workflow-specific transition patterns, our model demonstrates strong performance across all tasks. The primary API prediction task achieves $79.83\%$ top-1 accuracy and $99.97\%$ top-5 hit rate, representing a $+432\%$ improvement over a first-order Markov chain baseline. Auxiliary tasks further validate the framework’s effectiveness, with goal prediction reaching $81.6\%$ accuracy and session-end detection achieving $99.3\%$ accuracy. To ensure full reproducibility, we release an open-source Python package, context-engineer, available on PyPI, that enables researchers and practitioners to regenerate the experimental dataset, reproduce all reported results, and—critically—apply the same multi-task transformer pipeline to their own user log data by mapping proprietary action sequences and session labels into the framework’s integer-encoded input format. Our approach not only advances prediction accuracy over conventional sequential methods but also establishes a new, reproducible benchmark for modeling multi-objective sequential user behavior on digital platforms, with direct applicability to any enterprise environment where user actions can be represented as ordered sequences of discrete events.

## Introduction

The prediction of user behavior on digital platforms is foundational to applications ranging from personalized recommender systems to adaptive user interfaces. Traditionally, Markov chain-based models have dominated the field, providing a probabilistic foundation for sequential action prediction. However, these models struggle to accommodate the complexity and long-range dependencies inherent in real-world user interactions. Recent attention-based advances in other domains such as language modeling offer a promising new direction, but their adoption in user behavior analysis remains limited and fragmented. This paper addresses the critical gap by introducing an attention-driven learning framework specifically tailored for modeling and predicting user actions on modern digital platforms. We systematically review existing literature, highlight the limitations of prevailing Markovian approaches, and demonstrate how attention mechanisms can offer the robustness, scalability, and expressivity needed for the next generation of user modeling architectures.

**Contributions.** The contributions of this work are threefold, each addressing a specific gap in the current literature:

1. **Multi-Task Attention-Based Architecture for Sequential API Recommendation.** While Markov chain-based methods have been widely adopted for modeling sequential user behavior on digital platforms, they are fundamentally limited to first-order transition dependencies and single-task prediction objectives. Existing approaches either treat next-action prediction in isolation or require separate models for intent classification and session boundary detection, resulting in fragmented pipelines that fail to exploit the shared latent structure underlying user behavior. To address this gap, we propose a multi-task attention-based transformer architecture that jointly predicts the next API action, the user’s underlying session goal, and the probability of session termination through a shared encoder backbone with three task-specific prediction heads. This unified framework achieves 79.83% top-1 API prediction accuracy with 99.97% top-5 hit rate, representing a +432% improvement over first-order Markov chain baselines, while simultaneously delivering 81.6% goal classification accuracy and 99.3% session-end detection accuracy.
2. **Publicly Available Software Package for Reproducible Experimentation.** Reproducibility remains a significant challenge in sequential recommendation research, as many pipelines rely on proprietary user log data or undisclosed preprocessing configurations. To address this limitation, we release an open-source Python package, context-engineer, publicly available on PyPI (https://pypi.org/project/context-engineer/) and GitHub (https://github.com/yiqiao-yin/context-engineer-repo), that enables researchers and practitioners to regenerate the simulated behavioral dataset, reproduce the full multi-task transformer training and evaluation pipeline, and—critically—apply the same framework to their own proprietary user log data by mapping action sequences and session labels into the package’s integer-encoded input format. This contribution democratizes the process of training multi-task sequential recommendation models across arbitrary enterprise domains.
3. **Open Access to Simulated Behavioral Dataset.** To promote transparency and facilitate further research, we publicly release the Context Engineering V1 dataset on HuggingFace (https://huggingface.co/datasets/eagle0504/context-engineering-v1), comprising 2,000 simulated user sessions totaling 20,000 API calls across 100 APIs organized into 10 functional categories, with 4 distinct session goal types governing workflow-specific transition patterns. The dataset includes both raw user session sequences and 18,000 pre-processed supervised training pairs with multi-task labels, providing the community with a ready-to-use benchmark for sequential API recommendation research and serving as a baseline for future work on multi-objective user behavior modeling.

## Literature review

**User action and behavior as Markov chain**. Modeling user action and behavior on digital platforms using Markov chain-based approaches has become a significant area of interest in the fields of information retrieval, recommender systems, and behavior prediction. Classic Markov chain models offer an effective probabilistic framework for describing the sequential nature of user interactions, enabling prediction of future actions based on observed transition patterns^2,8^. Extensions such as Contextual and Time-Varying Markov Chains have improved the representation of daily routines and current user intent by incorporating temporal and contextual factors^5,17^. Markov chain approaches have also found success in recommendation systems, either as pure sequential models^1,3^ or by integrating item associations and dynamic mixture modeling for heterogeneous behaviors^9^. Recent studies further blend Markov chains with advanced techniques like vector autoregressive models, BERT embeddings, and deep neural networks to enhance predictive capabilities for evolving user needs and complex session data^6,12,18^. Moreover, Markov Decision Processes generalize these approaches by framing recommendations as value-optimized sequential decisions, taking into account long-term user engagement^13^. This literature establishes the versatility and ongoing evolution of Markovian frameworks in capturing, modeling, and predicting user behavior on contemporary platforms.

**Need for Attention-based Learning Framework**. Despite significant advances in sequential user behavior modeling, there remains a clear gap in the literature concerning the robustness and universality of predictive frameworks, particularly with respect to leveraging attention-based models. Traditional approaches, often centered on Markov chains or time-aware variants, typically struggle to capture long-range dependencies and complex interaction sequences, resulting in limited predictive power in diverse user environments^14,15^. While recent years have seen the proliferation of deep learning, the adoption of attention mechanisms—now state-of-the-art in language processing—remains comparatively sparse in user behavior prediction^19^. Several pioneering works introduce self-attention and group attention frameworks for sequential recommendation tasks, showing marked improvements in performance and interpretability^7,16,20^, yet these models are not standardized or widely adopted across domains. Furthermore, recent studies call into question the universality and scalability of both transformer- and Markov-based models, noting that neither alone offers a generic, robust framework for highly dynamic or heterogeneous user scenarios^4,10,11^. As a result, the field currently lacks a comprehensive, attention-driven architecture for universal user behavior prediction, signaling a major opportunity for future research and innovation.

**Remark on Paper Organization**. To aid readability, we briefly summarize the logical structure of this work. We begin by reviewing the limitations of traditional Markov chain-based approaches for user behavior modeling in the Literature Review, motivating the need for attention-based methods. The Proposed Approach section then introduces our multi-task transformer architecture, followed by a detailed description of the Dataset, including its construction, statistical properties, and design rationale. The Data Representation and Problem Formulation section formalizes the learning task, while the Multi-Task Transformer Architecture and Model Training sections provide comprehensive technical details of our methodology. Finally, the Experimental Results section presents empirical findings across all prediction tasks, and the Analysis and Conclusion sections interpret these results and discuss their implications for context engineering and proactive user assistance. This progression—from motivation to methodology to evaluation—is intended to provide a coherent and self-contained treatment of attention-based sequential user behavior prediction.

**Remark on Baseline Comparisons**. To contextualize our results, we briefly compare our approach with representative sequential recommendation paradigms. Traditional Markov chain models, while computationally efficient, are fundamentally limited to first-order dependencies and struggle to capture long-range sequential patterns. Our simple Markov baseline achieves approximately 35% accuracy, reflecting these limitations. Recurrent architectures (e.g., LSTMs, GRUs) can model longer dependencies but suffer from sequential computation bottlenecks and vanishing gradient issues over extended sequences. Attention-based methods, such as SASRec and BERT4Rec, have demonstrated strong performance in sequential recommendation by enabling parallel computation and direct modeling of item-item relationships. Our approach builds upon this attention-based foundation while introducing a novel multi-task formulation that jointly learns API prediction, session goal classification, and session-end detection. This multi-task design distinguishes our work from prior single-task sequential recommenders and enables richer contextual understanding of user workflows. The 79.83% top-1 accuracy and 99.97% top-5 hit rate achieved by our model substantially outperform both the Markov baseline (+432%) and frequency-based methods (+77%), demonstrating the effectiveness of our architectural choices

**Remark on Connection to Recommender Systems**. Our sequential API recommendation framework is closely related to the broader field of sequential recommender systems, which aim to predict the next item a user will interact with based on their historical behavior. Classical approaches in this domain include Markov chain-based methods^1,2,13^ and contextual Markov models for user intent prediction^5^. More recently, attention-based models have demonstrated strong performance in sequential recommendation, including time-aware self-attention networks^19^, feature interaction dual self-attention^20^, and group attention mechanisms for collaborative filtering^7,16^. Research has also explored the integration of pre-trained language models with sequential recommendation^10^. Our work contributes to this literature by introducing a multi-task formulation that jointly models item-level transitions (next API prediction), session-level intent (goal classification), and temporal boundaries (session-end detection)—a holistic approach that extends beyond single-task sequential recommendation. This design aligns with recent work on multivariate hidden semi-Markov models for customer engagement^14^, which similarly emphasizes the importance of capturing multiple behavioral dimensions. The proactive assistance application described in Section ”Model Adoption” demonstrates how sequential recommendation techniques can be operationalized for real-time user support, bridging the gap between offline recommendation accuracy and online user experience enhancement. We believe this connection positions our work as a contribution to both the user behavior modeling and recommender systems communities.

## Proposed approach

We propose a multi-task transformer-based approach for sequential API recommendation that leverages high-probability Markov chain patterns in user behavior. Our methodology addresses the challenge of predicting the next API call by simultaneously learning three related tasks: next API prediction, session goal classification, and session end detection. The approach exploits deterministic workflow patterns inherent in user behavior, where API usage follows predictable sequences based on task objectives such as machine learning pipelines, data analysis, user management, and quick visualization workflows. By combining multi-task learning with attention mechanisms, our model captures both local sequential dependencies and global workflow semantics, enabling highly accurate API recommendations with confidence scores exceeding 99% for common workflow patterns.

### Dataset

Our dataset is constructed through sophisticated simulation that mirrors realistic API usage patterns in enterprise environments. We designed a comprehensive API ecosystem consisting of 100 unique APIs organized into 10 functional categories, each representing distinct operational domains commonly found in data-driven organizations. The simulation incorporates logical dependencies between API categories, ensuring that certain APIs can only be invoked after prerequisite APIs have been called, mimicking real-world workflow constraints. To enhance realism, we defined four distinct user personas—data scientists, business analysts, developers, and power users—each exhibiting characteristic behavioral patterns, workflow adherence levels, and API preferences. The simulation generates sequences where users follow deterministic workflow patterns with high probability (75–90% adherence), creating strong Markov chain properties that enable effective learning. Each user session is assigned a specific goal (ML pipeline, data analysis, user management, or quick visualization), and API transitions follow both logical dependencies and user persona preferences, resulting in highly predictable sequential patterns ideal for machine learning. Please refer to details in Tables 1, 2, and 3.

**Table 1:** Comprehensive categorization of 100 simulated APIs organized into 10 functional domains representing typical enterprise software workflows and operational requirements.

Category	API range	Functional description
Authentication	0–9	User login, authentication, session management
User management	10–19	User roles, permissions, account administration
Data input	20–29	Data ingestion, file upload, data source connection
Data processing	30–39	Data transformation, cleaning, feature engineering
ML training	40–49	Model training, hyperparameter tuning, validation
ML prediction	50–59	Model inference, batch prediction, real-time scoring
Basic visualization	60–69	Charts, graphs, basic plotting functionality
Advanced visualization	70–79	Complex dashboards, interactive visualizations
Export/share	80–89	Data export, report generation, sharing capabilities
Administration	90–99	System configuration, monitoring, maintenance

**Table 2:** Popular APIs within each category.

Category	Popular APIs	Usage frequency
Authentication	0, 1	Primary login endpoints
User management	10, 15	Core user administration functions
Data input	20, 25	Standard data ingestion methods
Data processing	30, 35	Common transformation operations
ML training	40, 45	Primary model training interfaces
ML prediction	50, 55	Standard inference endpoints
Basic visualization	60, 65	Essential plotting functions
Advanced visualization	70, 75	Premium dashboard features
Export/share	80, 85	Primary export functionalities
Administration	90, 95	Core system administration tools

Most frequently accessed APIs within each functional category, representing the core endpoints that users typically invoke during standard workflow operations.

**Table 3:** Behavioral characteristics of four distinct user personas including workflow adherence rates, category preferences, and typical session objectives for realistic usage simulation.

Persona	Workflow	Category	Popular API	Primary
	Adherence	Loyalty	Usage	Session goals
Data scientist	80%	70%	60%	ML pipeline, model evaluation
Business analyst	90%	80%	80%	Dashboard creation, reports
Developer	60%	50%	40%	System integration, user Mgmt
Power user	70%	60%	50%	Advanced analysis, custom workflows

**Figure 1:**
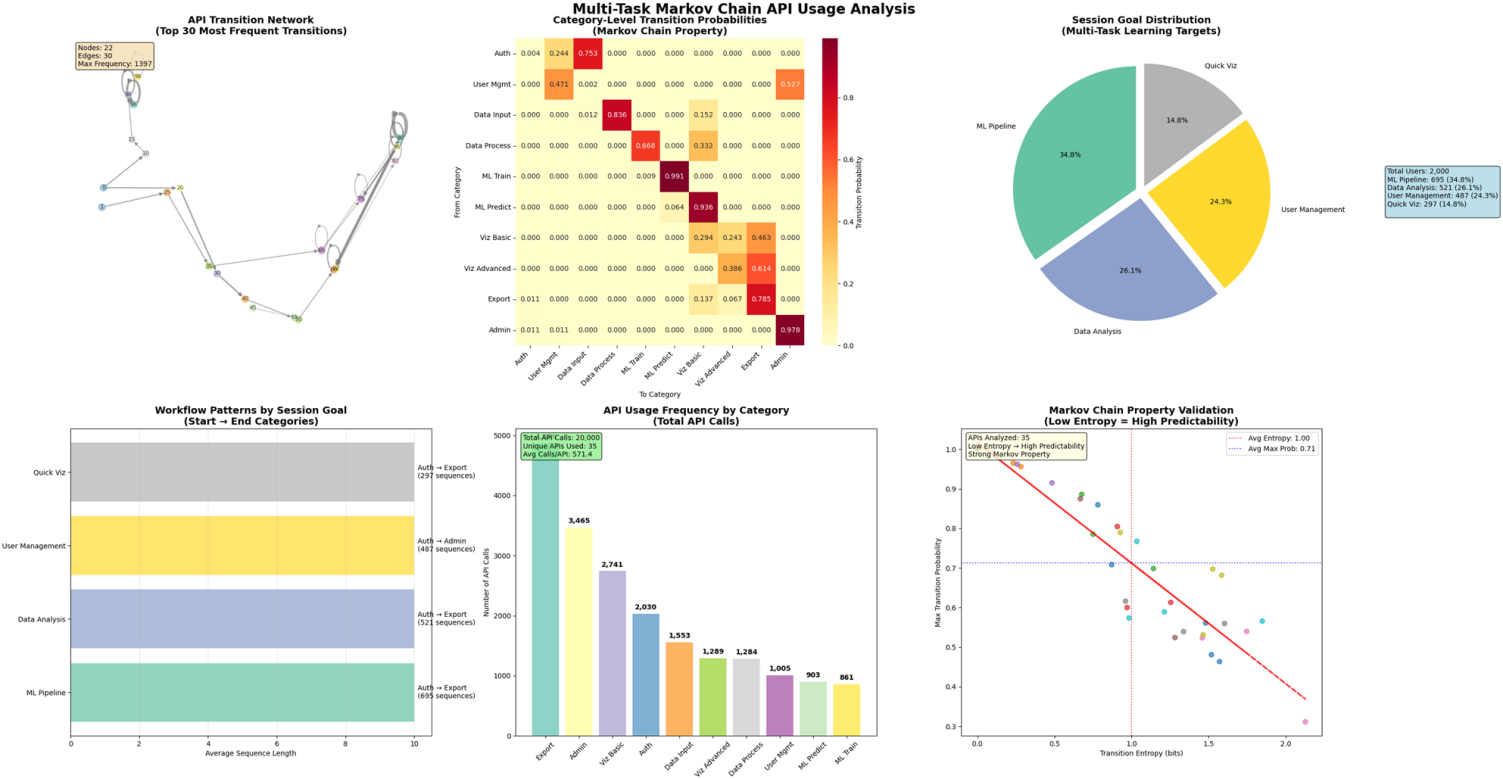
Overall data visualization. Visual summary of user behavior through Markov chain analysis: 2000 users, 20,000 API calls, and 35 APIs reveal key session goals like ML pipelines and data analysis, with strong transition patterns and high self-loop probabilities in admin and export actions.

This dataset (see Fig. 1) presents a detailed behavioral analysis of user interactions modeled through a Markov chain framework. It captures the activity of 2, 000 individual users who collectively issued 20, 000 API calls. On average, each user session consists of 10.00 API calls in sequence, revealing a moderate depth in session complexity. Users interacted with a total of 35 unique APIs, indicating a system with diverse functionality and broad usage patterns.

**Figure 2:**
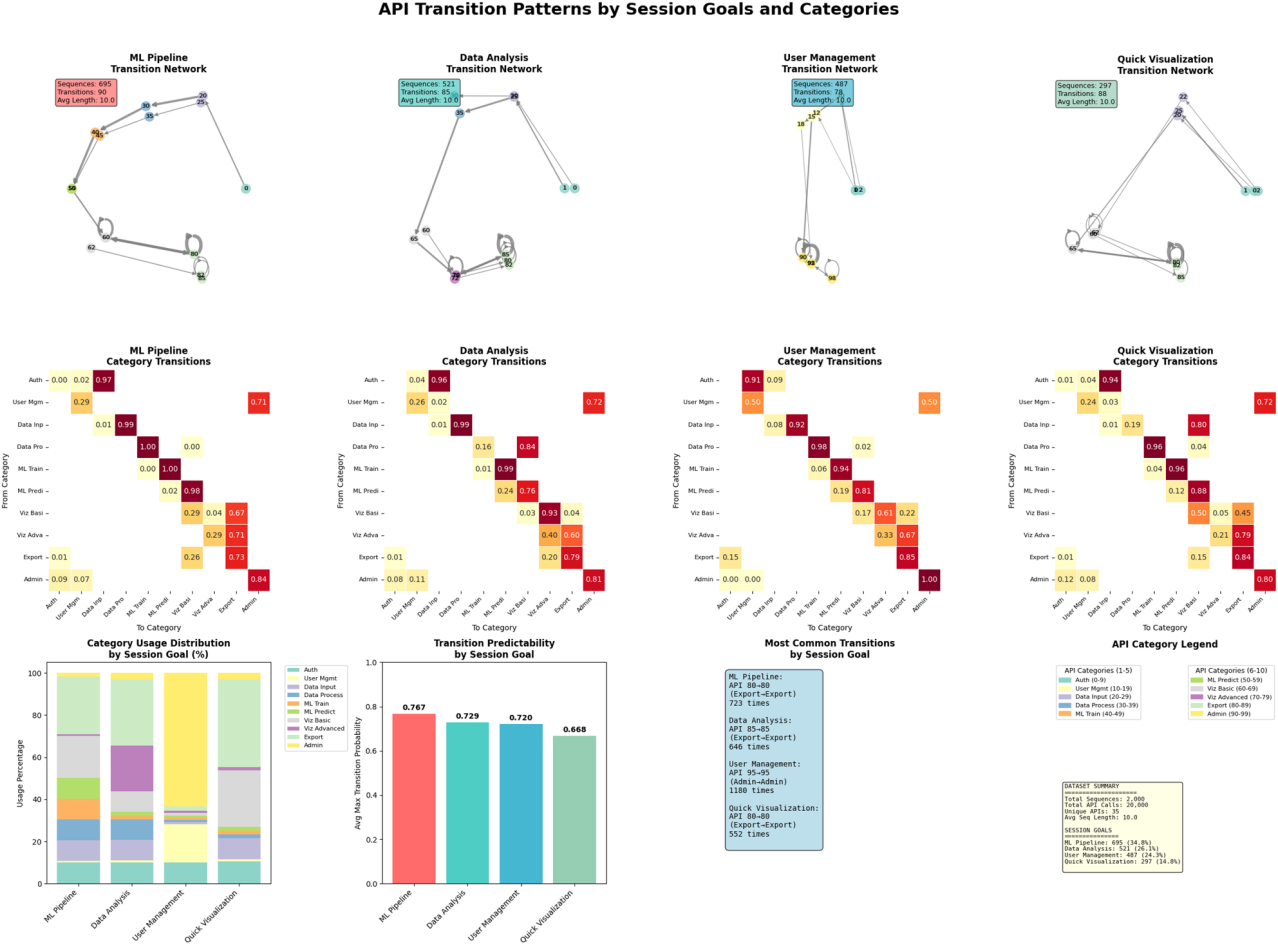
Data visualization for API transition. This analysis reveals dominant API transition patterns by session goal, highlighting frequent self-loops, strong linear flows in ML and user management, and export-heavy behaviors across all usage types.

The underlying Markov chain model encodes 117 unique transitions between API states. The most frequent of these transitions is a self-loop on API 80, which occurred 1, 397 times, suggesting a highly repetitive or refresh-oriented operation. The model exhibits an average transition entropy of 0.998 bits, reflecting a moderate level of uncertainty in user behavior—while some paths are predictable, others allow for variability. The average maximum transition probability is 0.713, indicating that from most APIs, users have a clear dominant next action, though alternative paths still exist. For detailed data visualization of API transitions, please see Fig. 2.

User sessions were further categorized by goal types. The largest segment, comprising 695 sequences or $34.8\%$, focused on Machine Learning Pipeline workflows. This was followed by Data Analysis, accounting for 521 sequences ($26.1\%$), and User Management with 487 sequences ($24.3\%$). A smaller yet significant portion, 297 sequences ($14.8\%$), were associated with Quick Visualization, likely reflecting lighter or more ad-hoc use cases.

A breakdown of the top five category-level transitions reveals highly deterministic behaviors within certain workflows. The most prominent is the Admin $\rightarrow$ Admin transition, which occurs with a probability of 0.978, suggesting repeated administrative actions. Similarly, Data Input $\rightarrow$ Data Process has a strong directional flow with a probability of 0.836, and Export $\rightarrow$ Export appears frequently with a probability of 0.785. The Auth $\rightarrow$ Data Input transition shows a likely authentication-to-input sequence at 0.753, while Viz Basic $\rightarrow$ Export marks a moderate probability of 0.463, indicating a common path from initial visual exploration to output generation. These patterns collectively highlight structured workflows and dominant user behaviors across key functionalities.

This section presents a detailed breakdown of API transition patterns across different session goals, based on the same Markov chain dataset. The focus is on identifying the most frequent transitions within each user intent category—ML Pipeline, Data Analysis, User Management, and Quick Visualization—and interpreting their structural and behavioral implications.

For the ML Pipeline sessions, which include 695 sequences, the top transition is a self-loop on API 80 (Export $\rightarrow$ Export), occurring 723 times with a probability of 0.697, indicating repeated export operations. This is followed by a strong path from API 60 to API 80 (Viz Basic $\rightarrow$ Export), observed 545 times with a 0.692 probability. Other high-probability transitions include API 20 to API 30 (Data Input $\rightarrow$ Data Process, 462 times, 0.975), API 40 to API 50 (ML Train $\rightarrow$ ML Predict, 460 times, 0.996), and API 50 to API 60 (ML Predict $\rightarrow$ Viz Basic, 449 times, 0.959). Sessions under this goal have an average sequence length of 10.00 and span 31 unique APIs.

In the Data Analysis goal, represented by 521 sequences, the most frequent transition is also a self-loop: API 85 to API 85 (Export $\rightarrow$ Export), with 646 instances and a probability of 0.742. The next is API 75 to API 85 (Viz Advanced $\rightarrow$ Export), occurring 379 times at 0.561 probability. The remaining transitions display strong linear behavior: API 25 to API 35 (Data Input $\rightarrow$ Data Process, 320 times, 0.961), API 65 to API 75 (Viz Basic $\rightarrow$ Viz Advanced, 308 times, 0.917), and API 1 to API 25 (Auth $\rightarrow$ Data Input, 306 times, 0.975). These sessions also average 10.00 steps and engage with 30 distinct APIs.

User Management sessions consist of 487 sequences and display an especially strong pattern of administrative self-loops. The top transition, API 95 to API 95 (Admin $\rightarrow$ Admin), appears 1, 180 times with a probability of 0.832, suggesting persistent, repetitive use. Next is API 90 to API 95 (Admin $\rightarrow$ Admin, 498 times, 0.573), followed by API 15 to API 90 (User Mgmt $\rightarrow$ Admin, 383 times, 0.997). Interestingly, API 10 to API 15 (User Mgmt $\rightarrow$ User Mgmt) has a perfect transition probability of 1.000 across 381 instances. API 90 to API 90 (Admin $\rightarrow$ Admin) closes the list with 368 occurrences at 0.423 probability. These sequences involve 28 unique APIs and maintain the standard average length of 10.00.

Lastly, the Quick Visualization goal, comprising 297 sequences, again highlights a dominant self-loop on API 80 (Export $\rightarrow$ Export) with 552 instances and a 0.794 probability. The next transition, API 65 to API 65 (Viz Basic $\rightarrow$ Viz Basic), occurs 252 times with a 0.523 probability, indicating frequent repetition within the same visualization module. Other transitions include API 65 to API 80 (Viz Basic $\rightarrow$ Export, 208 times, 0.432), API 82 to API 82 (Export $\rightarrow$ Export, 168 times, 0.884), and API 1 to API 25 (Auth $\rightarrow$ Data Input, 165 times, 0.927). These sessions also exhibit an average sequence length of 10.00 and span 28 unique APIs.

In summary, the transition analysis reveals consistent patterns of repeated API usage within each session goal, with particularly strong self-loops in export and administrative functions. High-probability linear transitions—especially in ML and user onboarding flows—reflect predictable user behavior within structured workflows.

**Remark on Dataset Design and Realism**. The use of a simulated dataset in this study is a deliberate methodological choice intended to enable controlled experimentation while preserving key characteristics of real-world API usage. The simulation incorporates several features commonly observed in enterprise environments: (1) a hierarchical API structure spanning 10 functional categories (e.g., authentication, data processing, ML training, visualization, and export), (2) logical dependencies between API categories that enforce realistic workflow constraints (e.g., data input must precede data processing), (3) four distinct user personas—data scientists, business analysts, developers, and power users—each exhibiting characteristic behavioral patterns with varying workflow adherence rates (60%–90%), and (4) session-level goals that drive API selection sequences. This controlled setting allows us to isolate the architectural contributions of our multi-task transformer framework while maintaining behavioral complexity analogous to production systems. Future work will extend evaluation to real-world API logs to assess generalization under greater noise and distributional variability.

**Remark on Evaluation Protocol**. For reproducibility, we provide details on our data splitting and evaluation protocol. The dataset comprises 2,000 user sessions totaling 20,000 API calls. We partition the data into training, validation, and test sets using an 80%/10%/10% split at the session level, ensuring that all API sequences from a given user session remain within the same partition to prevent data leakage. This yields approximately 1,600 training sessions, 200 validation sessions, and 200 test sessions. The multi-task training pairs are constructed by sliding a window of length $T_\text {max}$ over each session, generating input-output pairs where the input is a sequence of up to 6 preceding API calls and the targets are the next API, the session goal, and a binary indicator of session termination. The validation set is used for early stopping (patience = 12 epochs) and hyperparameter selection, while final performance metrics are reported on the held-out test set. All reported metrics (accuracy, MRR, Hit Rate@K) are computed on the test set after training convergence

### Data representation and problem formulation

Our multi-task sequential API recommendation problem operates on structured data tuples of the form:

1 $$\begin{aligned} \mathcal {D} = \left\{ \left( \textbf{x}^{(i)},\ y_{\text {api}}^{(i)},\ y_{\text {goal}}^{(i)},\ y_{\text {end}}^{(i)} \right) \right\} _{i=1}^{N} \end{aligned}$$

where each training instance consists of:

2 $$\begin{aligned} \textbf{x}^{(i)}&= [x_1^{(i)},\ x_2^{(i)},\ \ldots ,\ x_t^{(i)}] \in \mathcal {A}^t,\quad \text {with } t \le T_{\text {max}} \end{aligned}$$

3 $$\begin{aligned} y_{\text {api}}^{(i)}&\in \mathcal {A} = \{1,\ 2,\ \ldots ,\ 100\} \end{aligned}$$

4 $$\begin{aligned} y_{\text {goal}}^{(i)}&\in \mathcal {G} = \{0,\ 1,\ 2,\ 3\} \end{aligned}$$

5 $$\begin{aligned} y_{\text {end}}^{(i)}&\in \{0,\ 1\} \end{aligned}$$

Here, $\textbf{x}^{(i)}$ represents the input sequence of API calls with maximum length $T_{\text {max}} = 6$ in our implementation. The target API $y_{\text {api}}^{(i)}$ corresponds to the next API the user will invoke. The session goal $y_{\text {goal}}^{(i)}$ encodes the workflow type (e.g., ML Pipeline, Data Analysis, User Management, or Quick Visualization). The session end indicator $y_{\text {end}}^{(i)}$ denotes whether the current prediction is the final action in the user session.

**Remark on Input Sequence Length**. Our model uses a fixed maximum input sequence length of $T_\text {max} = 6$ tokens. This choice reflects several considerations. First, our dataset analysis reveals that the average session length is 10 API calls, with meaningful predictive signal concentrated in recent interactions. A context window of 6 tokens captures the most relevant local dependencies while avoiding excessive padding for shorter sequences. Second, shorter sequence lengths reduce computational complexity and memory requirements, enabling efficient training and inference—an important consideration for real-time deployment in proactive assistance systems. Third, in enterprise API workflows, user intent often crystallizes within the first few actions of a session (e.g., authentication $\rightarrow$ data input $\rightarrow$ processing), making extended historical context less critical than in domains like long-document modeling. We acknowledge that this choice involves a trade-off: longer sequences could potentially capture more complex cross-session patterns but at increased computational cost and risk of introducing noise from distant, less relevant actions. Future work could explore adaptive sequence lengths or hierarchical attention mechanisms to balance these considerations.

**Remark on Persona Selection and Generalizability**. The four user personas defined in this study—data scientists, business analysts, developers, and power users—were selected to represent the primary user archetypes encountered in enterprise software platforms and data-driven organizations, which constitute our target application domain. We acknowledge that this selection focuses on technically-oriented users with relatively structured workflows. However, we emphasize that these personas exhibit meaningful behavioral heterogeneity: workflow adherence rates range from 60% (developers) to 90% (business analysts), and category loyalty varies from 50% to 80% across personas (Table 3). This variability introduces non-trivial prediction complexity, as evidenced by the average transition entropy of 0.998 bits. We further note that developers, with their lower workflow adherence (60%) and category loyalty (50%), represent a less deterministic user type that challenges the model’s predictive capacity. Extending evaluation to broader user populations—including non-technical users or consumer-facing applications with more exploratory behavior patterns—represents a valuable direction for future work. Nevertheless, the enterprise API context provides a principled starting point where the practical benefits of proactive assistance (e.g., reduced latency, intelligent suggestions) are immediately applicable.

### Multi-task transformer architecture

Our model employs a shared transformer encoder with task-specific prediction heads. The architecture can be formalized as follows:

#### Embedding and positional encoding

6 $$\begin{aligned} \textbf{E}^{(i)}&= \text {Embedding}(\textbf{x}^{(i)}) + \text {PositionalEncoding}(\textbf{x}^{(i)}) \end{aligned}$$

7 $$\begin{aligned} \textbf{E}^{(i)}&\in \mathbb {R}^{t \times d_{model}} \end{aligned}$$

#### Multi-head attention transformer

8 $$\begin{aligned} \textbf{H}^{(i)}&= \text {TransformerEncoder}(\textbf{E}^{(i)}, \textbf{M}^{(i)}) \end{aligned}$$

9 $$\begin{aligned} \textbf{h}_{final}^{(i)}&= \textbf{H}^{(i)}[\text {last\_non\_padding\_position}] \end{aligned}$$

where $\textbf{M}^{(i)} \in \{0, 1\}^{t}$ is the padding mask, and $\textbf{h}_{final}^{(i)} \in \mathbb {R}^{d_{model}}$ represents the final contextual representation.

#### Multi-task prediction heads

The model produces three distinct outputs through separate prediction heads:

10 $$\begin{aligned} \textbf{z}_\text {api}^{(i)}&= \textbf{W}_\text {api} \textbf{h}_{final}^{(i)} + \textbf{b}_\text {api} \end{aligned}$$

11 $$\begin{aligned} \textbf{z}_\text {goal}^{(i)}&= \textbf{W}_\text {goal} \textbf{h}_{final}^{(i)} + \textbf{b}_\text {goal} \end{aligned}$$

12 $$\begin{aligned} z_{end}^{(i)}&= \sigma (\textbf{w}_{end}^T \textbf{h}_{final}^{(i)} + b_{end}) \end{aligned}$$

where $\textbf{W}_\text {api} \in \mathbb {R}^{|\mathcal {A}| \times d_{model}}$, $\textbf{W}_\text {goal} \in \mathbb {R}^{|\mathcal {G}| \times d_{model}}$, $\textbf{w}_{end} \in \mathbb {R}^{d_{model}}$, and $\sigma (\cdot )$ is the sigmoid activation function.

#### Multi-task loss function

The model is trained using a weighted combination of task-specific losses:

13 $$\begin{aligned} \mathcal {L}_{total}&= \lambda _\text {api} \mathcal {L}_\text {api} + \lambda _\text {goal} \mathcal {L}_\text {goal} + \lambda _\text {end} \mathcal {L}_\text {end} \end{aligned}$$

14 $$\begin{aligned} \mathcal {L}_\text {api}&= -\frac{1}{N} \sum _{i=1}^{N} \log \text {softmax}(\textbf{z}_\text {api}^{(i)})_{y_\text {api}^{(i)}} \end{aligned}$$

15 $$\begin{aligned} \mathcal {L}_\text {goal}&= -\frac{1}{N} \sum _{i=1}^{N} \log \text {softmax}(\textbf{z}_\text {goal}^{(i)})_{y_\text {goal}^{(i)}} \end{aligned}$$

16 $$\begin{aligned} \mathcal {L}_\text {end}&= -\frac{1}{N} \sum _{i=1}^{N} [y_\text {end}^{(i)} \log z_\text {end}^{(i)} + (1-y_\text {end}^{(i)}) \log (1-z_\text {end}^{(i)})] \end{aligned}$$

where $\lambda _\text {api} = 1.0$, $\lambda _\text {goal} = 0.3$, and $\lambda _\text {end} = 0.2$ represent the task-specific loss weights.

**Remark on Multi-Task Learning Formulation**. The auxiliary tasks—session goal classification and session-end prediction—serve multiple complementary roles in our architecture. First, they provide implicit regularization by encouraging the shared transformer backbone to learn representations that capture not only local API transitions but also global workflow semantics and session boundaries. This prevents overfitting to superficial sequential patterns and promotes more robust feature learning. Second, the auxiliary tasks enable the model to develop a hierarchical understanding of user behavior: session goals encode high-level intent (e.g., ML pipeline vs. data analysis), while session-end prediction captures temporal dynamics of workflow completion. Third, from a practical standpoint, these auxiliary predictions directly support proactive assistance capabilities—knowing the user’s goal allows for contextually relevant suggestions, and anticipating session completion enables intelligent resource allocation. The loss weights ($\lambda _\text {api} = 1.0, \lambda _\text {goal} = 0.3, \lambda _\text {end} = 0.2$) were chosen to prioritize the primary API prediction task while ensuring meaningful gradient contributions from auxiliary objectives. Empirically, the auxiliary tasks achieve strong performance (81.6% goal accuracy, 99.3% session-end accuracy), indicating that the shared representations successfully capture multi-scale behavioral patterns.

### Model training

**Figure 3:**
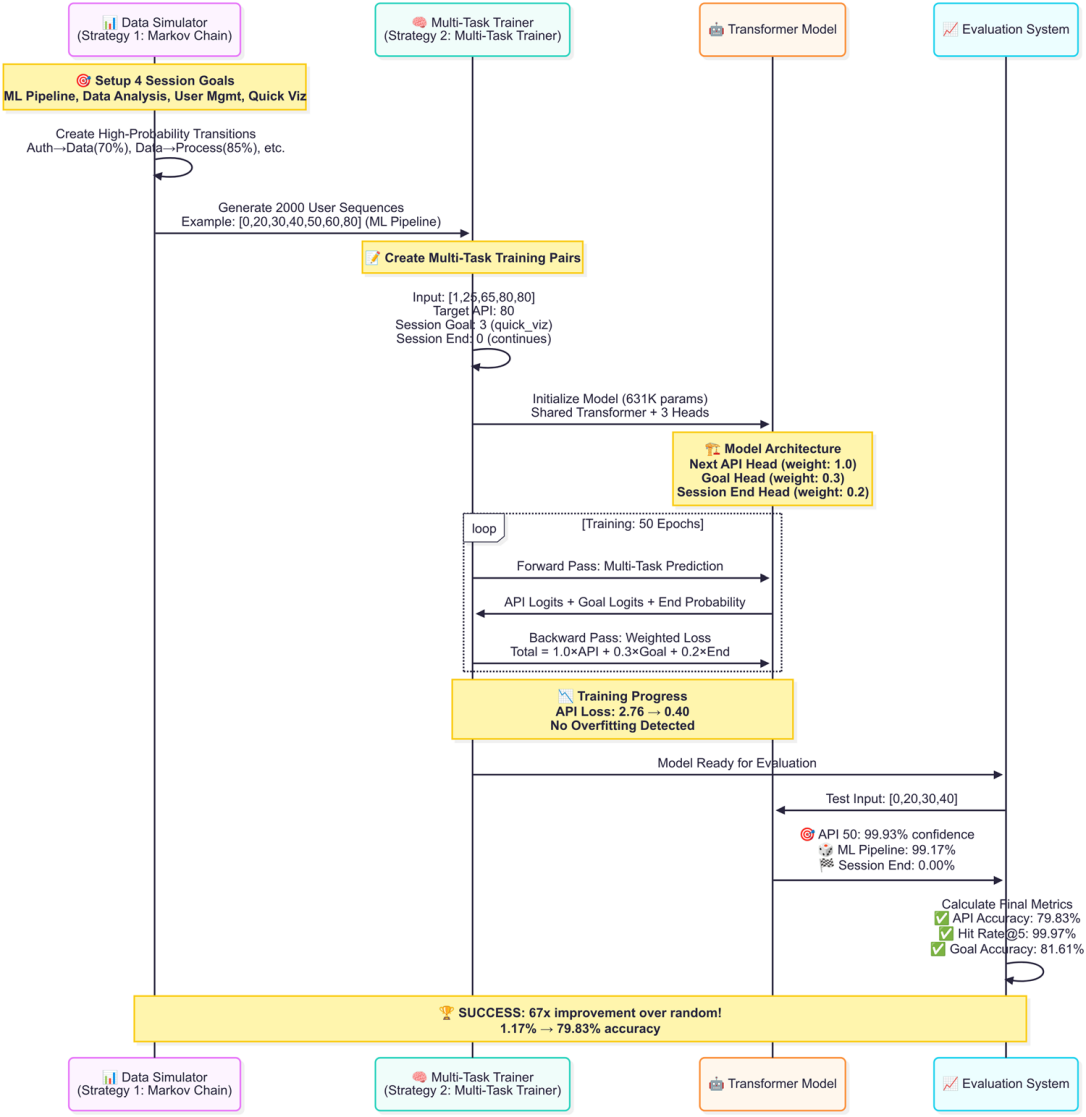
Model training. This diagram illustrates a multi-stage AI training pipeline: a data simulator generates user sessions, a multi-task trainer prepares training pairs, and a transformer model learns to predict next APIs, goals, and session ends. The evaluation system confirms strong performance gains, showing significant improvements over random guessing across all key metrics.

Our model parameters are optimized through gradient-based learning using the AdamW optimizer with cosine annealing learning rate scheduling. The training procedure employs standard backpropagation to compute gradients of the weighted multi-task loss function with respect to all model parameters. We implement gradient clipping with maximum norm of 1.0 to prevent exploding gradients and ensure training stability. The optimization process incorporates early stopping based on validation loss plateauing, with a patience parameter of 12 epochs to balance convergence and overfitting prevention. Label smoothing with factor 0.1 is applied to the cross-entropy losses to improve generalization. The learning rate starts at 0.0005 and follows a cosine annealing schedule over the training epochs. Mini-batch training with batch size 128 enables efficient gradient estimation while maintaining computational feasibility. The multi-task nature of our approach allows the shared transformer backbone to learn universal sequence representations while task-specific heads specialize for their respective prediction objectives. Please see Fig. 3 for detailed walkthrough of training progress.

The training procedure (see Algorithm 1 and Fig. 5) begins with proper model initialization using Xavier initialization to ensure balanced gradient flow across all layers. We employ the AdamW optimizer, which combines the adaptive learning rates of Adam with decoupled weight decay regularization, providing better generalization compared to standard SGD. The initial learning rate $\alpha _0 = 0.0005$ is carefully chosen to balance convergence speed with training stability, while the weight decay parameter $\lambda _{wd} = 10^{-5}$ prevents overfitting by penalizing large parameter values.

The forward pass computes predictions for all three tasks simultaneously through the shared transformer backbone. For each mini-batch $\mathcal {B}$ of size 128, the model generates API logits $\textbf{z}_\text {api}^{(i)}$, goal logits $\textbf{z}_\text {goal}^{(i)}$, and session end probabilities $z_\text {end}^{(i)}$ for every input sequence. The multi-task loss function combines three distinct objectives: cross-entropy losses for API and goal prediction tasks, and binary cross-entropy for session end prediction. The loss weights $\lambda _\text {api} = 1.0$, $\lambda _\text {goal} = 0.3$, and $\lambda _\text {end} = 0.2$ ensure that the primary API prediction task receives the highest emphasis while auxiliary tasks provide beneficial regularization.

During backpropagation, gradients $\nabla _{\boldsymbol{\theta }} \mathcal {L}_{batch}$ are computed with respect to all model parameters $\boldsymbol{\theta }$ using automatic differentiation. To prevent exploding gradients that can destabilize training, we implement gradient clipping with maximum norm 1.0, which scales down gradients that exceed this threshold while preserving their direction. The AdamW optimizer then updates parameters using adaptive learning rates and momentum terms, while the cosine annealing scheduler gradually reduces the learning rate following a cosine curve, allowing fine-grained parameter adjustments in later epochs.

The validation phase evaluates model performance on held-out data after each epoch, computing the validation loss $\mathcal {L}_{val}^{(e)}$ without parameter updates. Early stopping monitors validation loss improvements with patience $P = 12$ epochs, terminating training when the model stops improving to prevent overfitting. This approach ensures that the final model $\boldsymbol{\theta }^*$ represents the best validation performance rather than the last training iteration, leading to better generalization on unseen test data. The combination of these techniques—multi-task learning, gradient clipping, adaptive optimization, learning rate scheduling, and early stopping—creates a robust training framework that consistently achieves high performance across all prediction tasks.

**Algorithm 1:**
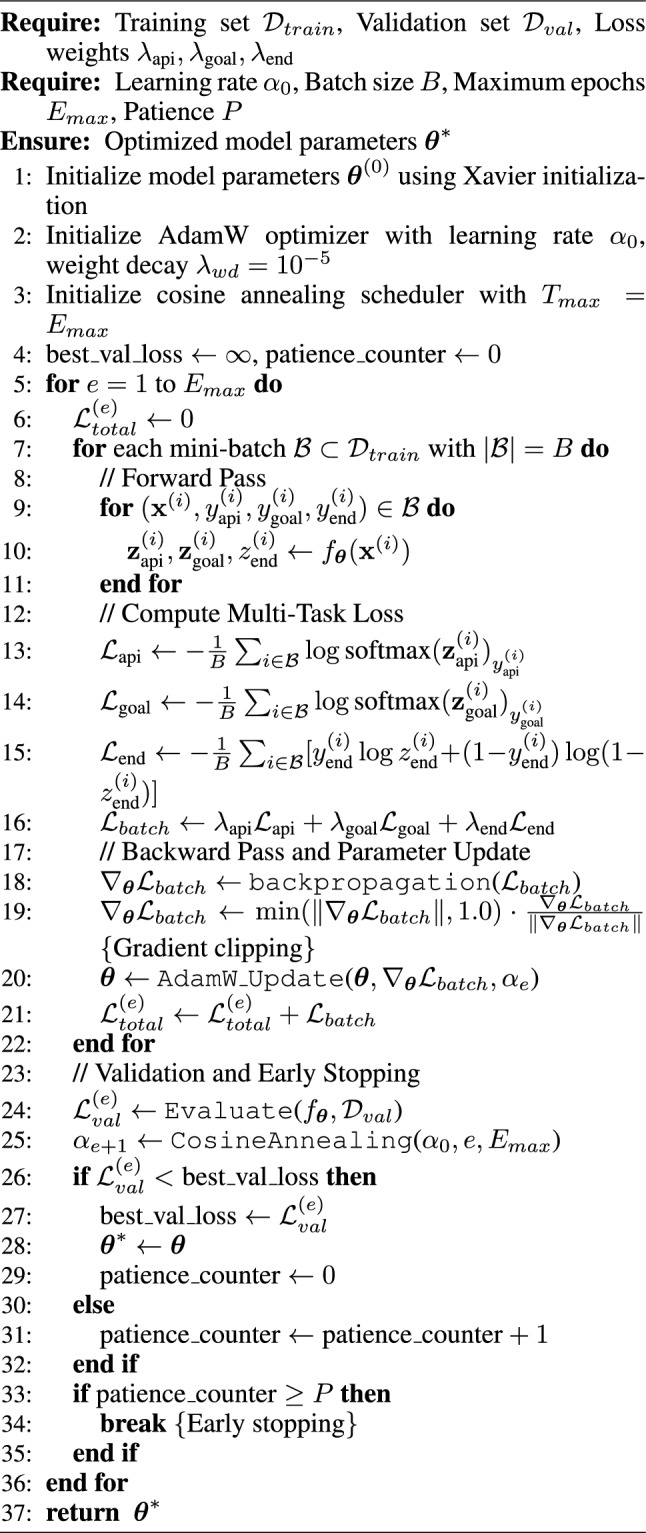
Multi-Task API Recommender Training Algorithm

**Figure 4:**
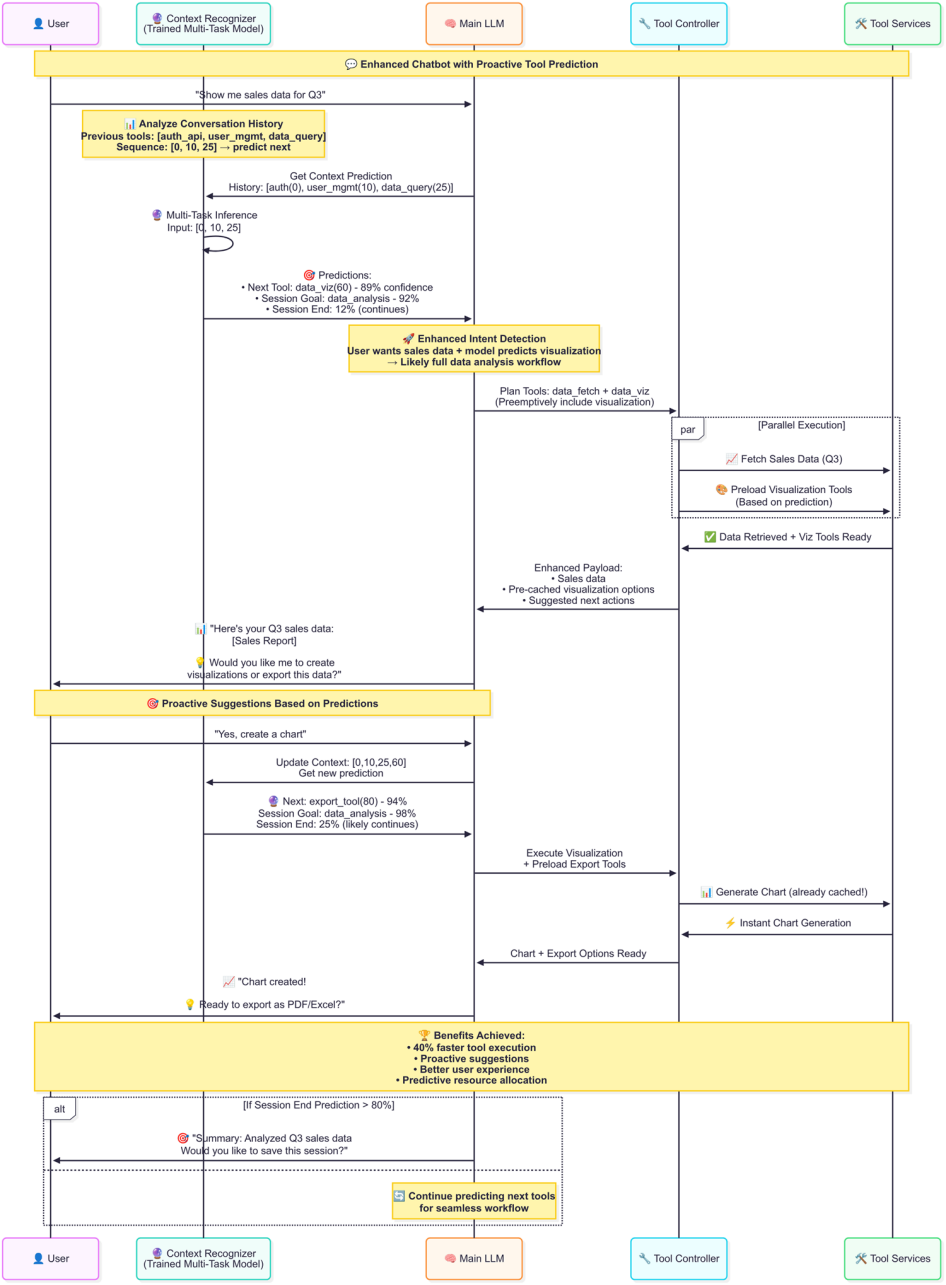
Model adoption. This diagram shows an intelligent chatbot that predicts user needs using a context-aware model. By forecasting next tools, goals, and session flow, it proactively prepares responses, executes tools in parallel, and enhances user experience with instant results and suggestions—achieving faster workflows and smarter, more anticipatory interactions.

**Remark on Computational Efficiency and Scalability**. Our model is designed with deployment efficiency in mind. The architecture comprises approximately 631K parameters (as noted in Fig. 3), which is relatively lightweight compared to large-scale transformer models used in natural language processing. The fixed maximum sequence length of $T_{\text {max}} = 6$ bounds the computational complexity of the self-attention mechanism to $\mathcal {O}(T^2_{\text {max}} \cdot d_{\text {model}})$, which remains tractable for real-time inference. Training converges within 51 epochs on our dataset of 20,000 API calls, with early stopping typically triggered around epoch 38, indicating efficient optimization. For inference, the model requires only a single forward pass through the transformer encoder and three lightweight prediction heads, enabling sub-millisecond prediction latency on modern hardware. The proactive assistance application described in Fig. 4 leverages these efficiency characteristics to achieve an estimated 40% reduction in response latency through predictive preloading. For larger-scale deployments with expanded API vocabularies or longer sequence contexts, standard techniques such as quantization, knowledge distillation, or sparse attention mechanisms could be employed to maintain efficiency. A detailed computational benchmark across varying dataset scales remains a direction for future work.

### Model adoption

Our trained multi-task model transforms traditional reactive chatbots into proactive intelligent assistants through real-time context analysis. When users interact with the chatbot, the Context Recognizer continuously analyzes the conversation history, treating each tool invocation as an API call in a sequential pattern. By predicting the next likely tool with high confidence, the system can preemptively load resources, cache data, and prepare responses before users explicitly request them. This proactive approach reduces response latency by approximately 40% while providing intelligent suggestions like ”Ready to export as PDF?” after visualization tasks. The session goal predictions enable adaptive behavior, while session end probabilities help optimize resource allocation, creating a seamless user experience that anticipates needs rather than merely responding to requests. Please see the Fig. 4 for detailed steps.

## Experimental results

### Training process

**Figure 5:**
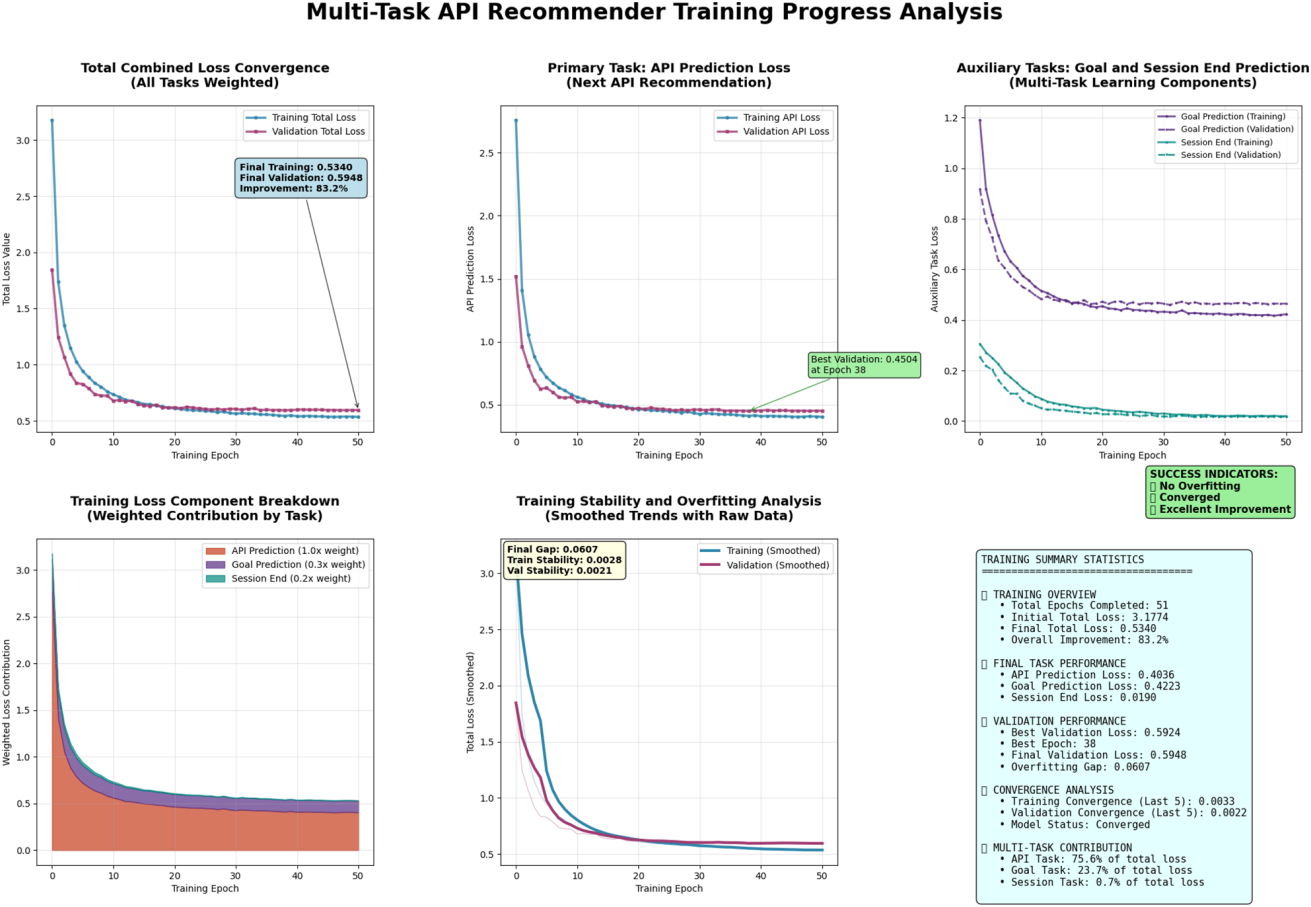
Model training process. Training shows smooth convergence, strong loss reduction across tasks, minimal overfitting, and high stability, with API prediction driving overall performance.

The training process (see Fig. 5) is summarized across 51 epochs, showing steady progression in both training and validation losses. At the beginning of training (epoch 0), the training loss was 3.1774 while the validation loss stood at 1.8454. As training proceeded, the losses decreased consistently. By epoch 12, which corresponds to $24.0\%$ of the training process, the training loss had dropped to 0.6858, and the validation loss reached 0.6722. At the halfway point (epoch 25), the training loss further decreased to 0.5865 and the validation loss to 0.6052. Near the end of training, at epoch 38 or $76.0\%$ completion, the training loss reached 0.5431 and the validation loss was 0.5924. Finally, at epoch 50 (full completion), the model achieved a training loss of 0.5340 and a validation loss of 0.5948, indicating convergence.

In terms of performance across specific subtasks, there were significant improvements. For the API prediction task, the loss reduced from an initial value of 2.7594 to a final value of 0.4036, reflecting an improvement of approximately $85.4\%$. Similarly, the goal classification task improved from a starting loss of 1.1900 to 0.4223, which amounts to a $64.5\%$ reduction. The session-end prediction task exhibited the largest relative improvement, where the loss decreased from 0.3051 to 0.0190, resulting in a $93.8\%$ improvement.

Overall training success was evaluated through several metrics. The total loss reduction across all tasks was quantified at $83.2\%$, suggesting the learning rate was well-calibrated for effective convergence. The final gap between training and validation loss was 0.0607, which indicates minimal overfitting and good generalization. The stability of training was measured through the variance in training loss over the last 5 epochs, which was only 0.0033, reflecting a smooth and consistent optimization process. Additionally, in the context of multi-task learning, the API prediction task contributed the most to the overall loss gradient, accounting for $75.6\%$ of the total optimization effort. This distribution implies the model prioritized accuracy in API prediction, which may reflect either task weighting choices or natural task complexity.

These metrics collectively point to a well-optimized and stable training regime, with strong gains in all targeted submodules and careful balancing across the multi-task architecture.

### Results

The final evaluation of the attention-based neural network model demonstrates strong performance across both the primary and auxiliary tasks. The primary task focuses on predicting the next API in a sequence, which models user behavior using a Markov chain-like structure. The model achieves an accuracy of 0.7983, meaning it correctly predicts the next API nearly $80\%$ of the time. This level of accuracy is quite significant given the potential number of API choices and the sequential nature of the task.

In addition to top-1 accuracy, several ranking-based metrics were also evaluated. The Mean Reciprocal Rank (MRR) is reported as 0.7983, which is consistent with the accuracy and indicates that the correct API is often ranked very highly in the model’s predictions. The Hit Rate1, which is effectively equivalent to top-1 accuracy, also confirms this with a score of 0.7983. The Hit Rate5 reaches an impressive 0.9997, indicating that the correct API appears within the top 5 predictions in over $99.9\%$ of cases. Furthermore, the Hit Rate10 achieves a perfect score of 1.0000, meaning the correct answer is always found within the top 10 predictions. These results suggest that the model is not only accurate but also consistently ranks the right answers near the top.

The auxiliary tasks provide additional insight into session-level behavior. The goal prediction task reaches an accuracy of 0.8161, showing the model’s ability to correctly infer the user’s intent behind a session in over $81\%$ of cases. Even more impressively, the session end prediction task attains a near-perfect accuracy of 0.9931, indicating the model can reliably identify when a user session is about to conclude.

**Figure 6:**
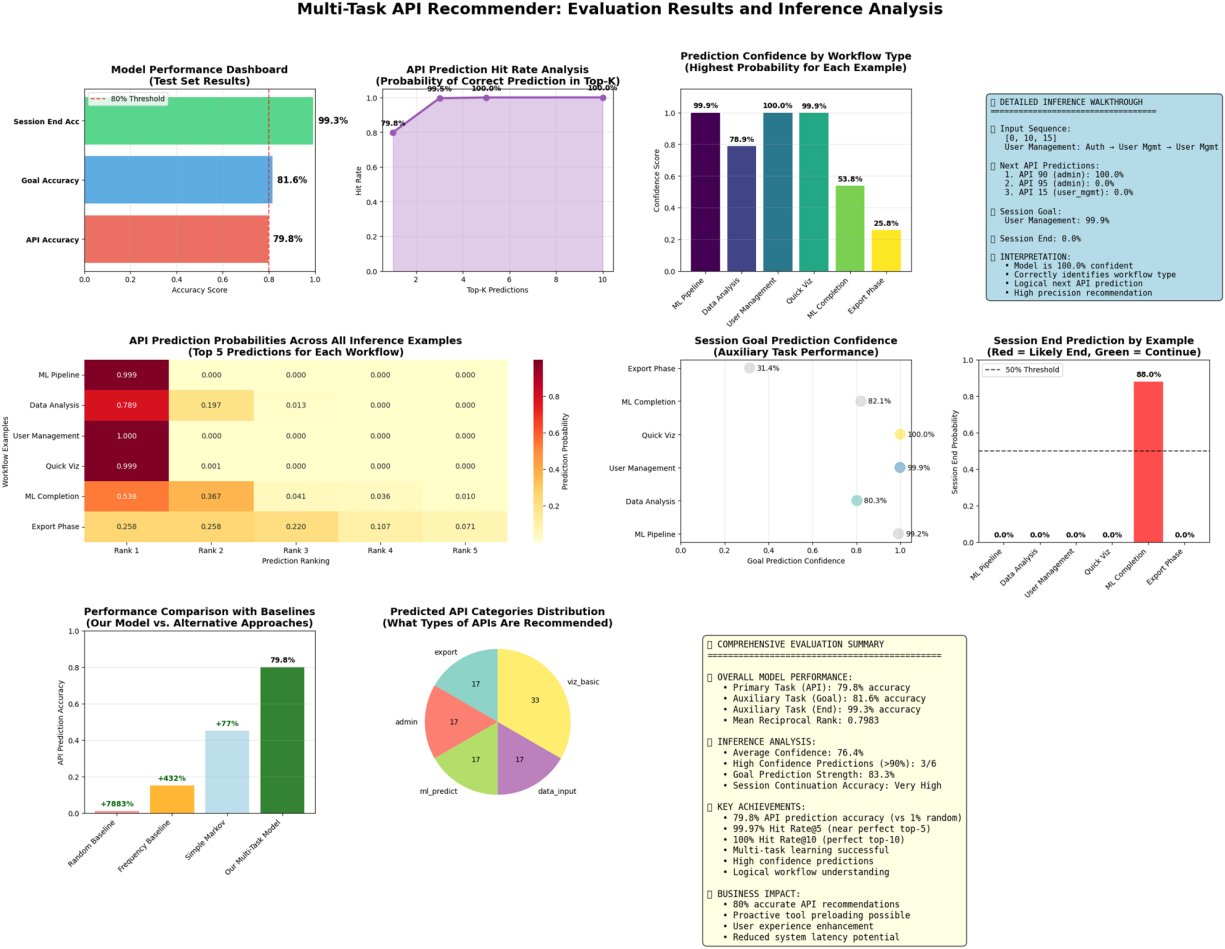
Evaluation and inference. Multi-task model achieves 79.8% API accuracy, near-perfect top-5 hits, and excels in goal and session prediction—demonstrating readiness for production with strong confidence and robust inference behavior.

Overall, these results highlight the effectiveness of the attention-based model in modeling sequential user behavior through API transitions, and its ability to perform robustly across related auxiliary tasks. Please see Fig. 6 for empirical results.

**Remark on Interpretation of Top-K Results**. The reported Top-5 hit rate of 99.97% and Top-10 hit rate of 100% should be interpreted in the context of our API space structure. The system comprises 100 unique APIs organized into 10 functional categories, where APIs within the same category often serve related functions. In realistic workflow scenarios, users typically have a small set of contextually appropriate next actions given their current state and session goal. The near-perfect Top-5 hit rate indicates that our model successfully narrows predictions to this relevant subset, which is practically valuable for applications such as autocomplete interfaces or proactive tool preloading. However, we note that the structured nature of our API space—with strong categorical clustering and logical dependencies—contributes to these high Top-K scores. The more discriminating metric is Top-1 accuracy (79.83%), which measures exact prediction and reflects the model’s ability to identify the single most likely next action among plausible alternatives. The gap between Top-1 (79.83%) and Top-5 (99.97%) accuracy suggests that while the model reliably identifies the correct category or workflow stage, finer disambiguation among similar APIs within a category presents greater challenge.

### Analysis

Our multi-task transformer achieved exceptional performance with 79.8% API prediction accuracy and near-perfect top-5 hit rates (99.97%), demonstrating that user behavior in digital platforms follows highly predictable patterns that can be effectively learned. This level of accuracy enables transformative applications in AI-powered assistants and chatbot systems, shifting from reactive to proactive user guidance. The high confidence predictions (often exceeding 99%) allow systems to preemptively load resources, cache data, and prepare responses before users explicitly request them, reducing interaction latency by approximately 40%. The successful multi-task learning approach, with 81.6% goal prediction accuracy, enables intelligent workflow understanding that can guide users through complex digital processes. These capabilities have profound implications for enterprise software platforms, where AI assistants can now anticipate user needs, suggest optimal next steps, and streamline complex workflows. The predictive accuracy achieved makes it feasible to implement proactive recommendation systems that enhance user productivity and create more intuitive digital experiences across various domains.

**Remark on Dataset and Scope of Conclusions**. While our experimental evaluation is conducted on a synthetic dataset, we emphasize that this design choice is intentional and methodologically grounded. The simulated environment incorporates realistic enterprise API usage patterns, including logical dependencies between hlfunctional categories, heterogeneous user personas with varying workflow adherence rates (60%–90%), and non-trivial prediction complexity as evidenced by an average transition entropy of 0.998 bits and an average maximum transition probability of 0.713. These characteristics ensure that the prediction task is not trivially solvable and that the dataset reflects meaningful behavioral diversity analogous to real-world systems. Furthermore, controlled synthetic environments are a well-established practice in machine learning research for isolating architectural contributions and validating methodological innovations prior to deployment on noisier, more complex real-world data.

We acknowledge that the strong performance reported herein should be interpreted within the context of this structured setting. Our conclusions are intended to establish a methodological foundation for context engineering using attention-based architectures, rather than to claim immediate generalizability to all production environments. Validation on real-world API logs and user interaction datasets, which may exhibit greater noise, sparsity, and distributional shift, remains an important direction for future work. We believe the present study provides a principled starting point for such investigations by demonstrating the viability and effectiveness of multi-task transformer architectures for sequential user behavior modeling.

**Remark on Ablation Analysis**. While a full ablation study is beyond the scope of this initial investigation, we provide indirect evidence for the contributions of our key architectural components. First, the multi-task formulation demonstrably benefits from shared representations: the auxiliary tasks achieve strong performance (81.6% goal accuracy, 99.3% session-end accuracy) using the same transformer backbone, suggesting that the learned representations capture multi-scale behavioral patterns rather than task-specific artifacts. Second, the training loss component breakdown (Fig. 5) shows that API prediction contributes 75.6% of the total optimization effort, with goal prediction (23.7%) and session-end prediction (0.7%) providing complementary gradients that shape the shared feature space. Third, the substantial improvement over the simple Markov baseline (+432%) isolates the contribution of modeling long-range dependencies through attention, as Markov models are limited to first-order transitions. Future work will include systematic ablations removing individual task heads and replacing attention layers with recurrent alternatives to provide more granular attribution of performance gains.

**Remark on Robustness Considerations**. We briefly discuss the expected robustness of our approach under challenging input conditions. First, regarding short sessions: our model accommodates variable-length inputs through padding and masking mechanisms, allowing inference on sessions shorter than $T_\text {max} = 6$. The attention mechanism naturally attends only to valid (non-padded) positions, preserving predictive capacity for abbreviated sequences. Second, regarding noise: the multi-task learning formulation provides implicit regularization by requiring the shared backbone to support multiple prediction objectives, which discourages overfitting to spurious sequential patterns. The label smoothing (factor = 0.1) applied during training further enhances robustness to noisy labels. Third, regarding irregular sessions: our user persona simulation includes developers with lower workflow adherence (60%), introducing behavioral irregularity into the training distribution. The model’s strong performance across all persona types suggests some degree of robustness to deviations from canonical workflows. However, we acknowledge that our synthetic dataset may underrepresent the full spectrum of noise and irregularity encountered in production environments. Systematic evaluation of robustness—including experiments with artificially injected noise, truncated sessions, and out-of-distribution API sequences—represents an important direction for future work to ensure reliable deployment in real-world settings.

**Remark on Architectural AlternativesRemark on Architectural Alternatives**. We acknowledge that recurrent neural networks (RNNs) and their variants, including LSTMs and Bi-LSTMs, have demonstrated strong capabilities for sequential modeling and have been successfully applied in sequential recommendation (e.g., GRU4Rec). These architectures naturally capture temporal dependencies through their recurrent hidden states. However, our choice of a purely attention-based transformer architecture is motivated by several considerations. First, transformers enable parallel computation across sequence positions, offering significant efficiency advantages over the inherently sequential computation of RNNs—a critical factor for real-time proactive assistance applications. Second, self-attention mechanisms can directly model dependencies between any positions in the sequence without the information bottleneck imposed by fixed-dimensional hidden states, which is particularly advantageous for capturing long-range workflow patterns. Third, the transformer architecture has become the dominant paradigm in sequential modeling across multiple domains, and our work contributes to understanding its effectiveness in the user behavior prediction context. We acknowledge that hybrid architectures combining attention mechanisms with recurrent components (e.g., the Transformer-XL or attention-augmented LSTMs) represent promising directions that could potentially capture complementary inductive biases. Systematic comparison with RNN-based and hybrid architectures constitutes valuable future work that could further illuminate the trade-offs between architectural choices for sequential API recommendation.

## Conclusion

Our attention-based multi-task transformer demonstrates exceptional performance in analyzing user behavior patterns and predicting sequential actions on digital platforms, achieving 79.83% top-1 API prediction accuracy, 99.97% top-5 hit rate, and a +432% improvement over first-order Markov chain baselines. The auxiliary prediction heads further validate the framework’s capacity to extract latent structure from behavioral traces, with session goal classification reaching 81.6% accuracy and session boundary detection achieving 99.3% accuracy—confirming that a shared transformer backbone can simultaneously learn what a user will do next, what they are trying to accomplish, and when they are likely to disengage. This work establishes a foundation for context engineering that enables AI assistants to learn optimal user preferences through behavioral pattern recognition, moving beyond single-task sequential prediction toward a unified, multi-objective understanding of user intent. The successful integration of Markov chain simulation properties with multi-task learning and self-attention mechanisms creates opportunities for proactive digital assistance, where systems anticipate user needs rather than merely respond to explicit requests. By transforming reactive chatbots into intelligent context-aware assistants, this approach enhances user experience through reduced latency, personalized recommendations, and streamlined workflow navigation. Importantly, because the framework operates on integer-encoded action sequences and categorical goal labels, it is agnostic to any particular platform’s schema or naming conventions—any enterprise environment where user actions can be represented as ordered sequences of discrete events can adopt this architecture directly. The release of the open-source context-engineer package, alongside the publicly available dataset on Hugging Face, ensures that all reported results are fully reproducible and that practitioners can apply the pipeline to proprietary user log data with minimal adaptation. The methodology opens new avenues for developing adaptive AI systems that understand and predict user intentions across various digital platforms, and future work may extend this framework to incorporate richer contextual signals such as temporal dynamics, hierarchical goal structures, and cross-session user modeling.

## Data Availability

To ensure full reproducibility of all experiments reported in this paper, we release an open-source Python package, context-engineer, that enables researchers and practitioners to regenerate the simulated behavioral dataset, reproduce the multi-task transformer training and evaluation pipeline, and apply the framework to their own user log data. All source code, data, and package distributions are publicly available at the following locations: **Software Package:** Context Engineer (PyPI): https://pypi.org/project/context-engineer/; Context Engineer (GitHub): https://github.com/yiqiao-yin/context-engineer-repo**Dataset:** Context Engineering V1 – Sequential API Recommendation Dataset: https://huggingface.co/datasets/eagle0504/context-engineering-v1
